# A High-Precision Anti-Jamming Algorithm Based on Newton-Iteration-Enhanced Three-Spectral-Line RIFE with Real-Time Implementation

**DOI:** 10.3390/s26113549

**Published:** 2026-06-03

**Authors:** Xinhua Tang, Yiming Wang

**Affiliations:** 1 Key Laboratory of Micro-inertial Instrument and Advanced Navigation Technology of Ministry of Education, Southeast University, Nanjing 210096, China; 220233668@seu.edu.cn; 2 State Key Laboratory of Comprehensive PNT Network and Equipment Technology, Southeast University, Nanjing 210096, China

**Keywords:** GNSS, anti-jamming, FPGA, FFT spectrum analysis, frequency estimation

## Abstract

GNSS signals are extremely weak at the Earth’s surface and are highly vulnerable to in-band interference, particularly high-dynamic linear frequency-modulated (LFM) jamming, which may lead to receiver loss of lock. Existing anti-jamming techniques struggle to balance real-time constraints with high-precision frequency estimation. This paper proposes a Newton-iteration-enhanced three-spectral-line RIFE algorithm implemented on a heterogeneous FPGA platform (Zynq-7000 SoC). The method performs coarse frequency estimation using the three-spectral-line RIFE to mitigate FFT fence effects, followed by Newton-based quadratic refinement, enabling high estimation accuracy with reduced FFT size. A fast–slow loop architecture is adopted, where the FPGA (PL) performs real-time interference suppression and the ARM (PS) handles system control and parameter updates. Experimental results show that, under static interference, the proposed method achieves a 10.9 dB improvement over direct estimation algorithms. Under chirp interference, it significantly outperforms both direct estimation and conventional iterative methods. In GNSS closed-loop tests, the proposed approach extends the anti-jamming margin to 82 dB J/S. Overall, the proposed method effectively balances estimation accuracy and processing latency, providing a practical solution for GNSS anti-jamming in high-dynamic environments.

## 1. Introduction

Global Navigation Satellite System (GNSS) provides all-weather, high-precision navigation and timing services, which are widely deployed across critical sectors such as national defense, transportation, power grid management, and financial communications. However, due to the long-distance space propagation from medium-to-high Earth orbits (approximately 20,000 km), GNSS signals arrive at ground receivers with extremely low power levels, typically around −130 dBmW. Although GNSS incorporates strategies like spread-spectrum processing to improve processing gain and enhance signal resilience, these techniques cannot completely eliminate the inherent vulnerability of the signals. When the strength of an interference signal exceeds the receiver’s anti-jamming tolerance margin, the front-end Automatic Gain Control (AGC) circuit may saturate, or the sharp degradation in the carrier-to-noise ratio may cause the tracking loops to lose lock. This ultimately leads to the failure of the receiver’s positioning and timing functions. Relevant studies have shown that even low-power, compact jamming devices can easily disrupt unprotected GNSS receivers within a 6 to 9 km radius.

**Figure 1:**
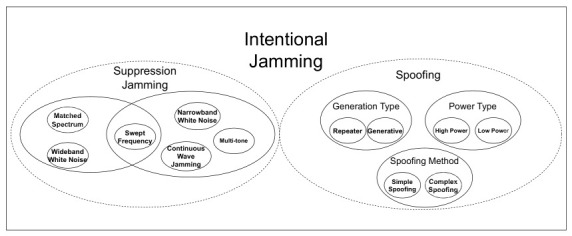
Types of jamming.

With the continuous advancement of electronic countermeasure (ECM) technologies, the electromagnetic environment where GNSS operates has become increasingly complex. Beyond unintentional radio frequency (RF) interference, intentional interference constitutes the primary threat. Intentional interference is typically categorized into two main types, jamming and spoofing, as illustrated in Figure 1.

Among the aforementioned interference types, jamming has emerged as the dominant form of interference due to its straightforward implementation and pronounced disruptive effects. Based on the interference bandwidth, jamming can be further categorized into narrowband and wideband interference. Narrowband jamming typically includes single-tone and multi-tone jamming, while wideband jamming primarily includes matched-spectrum and sweep-frequency jamming. Among these, Narrowband Interference and sweep-frequency interference are the most prevalent types.

Narrowband Interference (NBI) primarily manifests as single-tone or narrowband signals, with its energy concentrated at specific frequencies. Although NBI is relatively easy to detect, its high energy concentration can severely distort the signal’s spectral structure, resulting in significant degradation of the receiver’s carrier-to-noise ratio (C/N0). Conversely, Sweep-Frequency Interference is a highly dynamic, non-stationary signal whose frequency varies rapidly over time in either a linear or nonlinear manner. Compared to static NBI, sweep-frequency interference is capable of traversing the entire GNSS signal bandwidth, leading to sever disruption on both the acquisition and tracking stages of the receiver.

In the domain of GNSS anti-jamming techniques, the deployment of digital notch filters—particularly Infinite Impulse Response (IIR) notch filters—is widely recognized as the most prevalent and effective countermeasure against Narrowband Interference. The performance of the notch filter highly relies on the mismatch between its null frequency and the jamming center frequency; thus, the effectiveness of this suppression technique is strictly dependent upon the accuracy of the jamming frequency estimation. Even a marginal deviation in the estimated frequency can result in significant residual jamming energy leakage into the system, thereby severely degrading the receiver’s performance. Therefore, ultra-precise and real-time frequency estimation is a critical prerequisite for the successful implementation of notch-based interference mitigation techniques.

Although traditional Maximum Likelihood Estimation (MLE) provides high frequency estimation accuracy, it entails substantial computational overhead that hinders real-time implementation. In contrast, simple Fast Fourier Transform (FFT) operation is computationally efficient, but is fundamentally limited by the picket-fence effect, which restricts high-precision frequency estimation. To address this challenge, various frequency offset estimation algorithms based on interpolation, curve fitting, and iterative optimization have been proposed.

One important optimization direction is based on RIFE. Wang, X. et al. proposed a shifted RIFE algorithm that forcibly moves the spectral peak intensity to the intermediate region between two quantized frequency points via a precise frequency-domain shift [1]. This mechanism effectively avoids the worst-case spectral leakage scenarios inherent in conventional RIFE algorithms when the true frequency lies exactly halfway between two bins. To further enhance estimation performance under severe low SNR conditions, J. Wang et al. proposed a multi-point RIFE algorithm utilizing three or more spectral lines for weighted interpolation [2], successfully decreasing the variance of the estimate by leveraging adjacent side-lobe information. Niu et al. proposed a three-line interpolation algorithm that compensates for the main-lobe broadening of commonly used windows via advanced polynomial fitting [3]. For pulse signals, Jia Luo et al. designed a cascaded architecture that combines M-Rife for coarse estimation with Maximum Likelihood Estimation (MLE) for fine calibration [4]. This approach significantly reduces the search space of the MLE, achieving an absolute accuracy better than 0.3 Hz without exhaustive searching. Furthermore, Cheng et al. introduced an improved algorithm based on Phase Angle Interpolation (PAI-Rife) [5]; with the introduction of the frequency deviation factor, this method mitigates phase wrapping errors and improves estimation linearity. Jia et al. innovatively proposed the SP-Rife algorithm, which utilizes Spectral Refinement (Zoom-FFT) as a digital down-converter to isolate the target band before applying phase angle interpolation [6], demonstrating exceptional robustness and resolution even under heavy noise.

Another important refinement direction is based on the Quinn algorithm. The Quinn algorithm utilizes complex spectral line phase relationships for frequency estimation, but it inherently lacks robustness at low SNRs. To resolve this, Liu et al. proposed an improved Quinn algorithm utilizing amplitude and phase interpolation alongside a segmented spectrum-shifting strategy [7]. This method avoids the nonlinear distortion near the Nyquist frequency and DC components, reducing computational complexity while maintaining high fidelity. X. Zhang et al. proposed a joint estimation scheme cascading the frequency-domain Quinn algorithm with time-domain pilots [8], effectively resolving the phase ambiguities that purely frequency-domain methods often encounter. To enhance accuracy in specific high-resolution scenarios such as FMCW radar ranging, Duan et al. [9] and Fernando M. Janeiro [10] explored cascaded refinement schemes integrating the Fast Chirp-Z Transform (CZT) with the Quinn algorithm, where CZT is leveraged to “zoom in” on a target frequency band, and arbitrary resolution can be achieved without the substantial memory overhead associated with an N-point FFT. Under non-ideal channel conditions, Xie et al. adopted a minimax nonlinear transformation design [11], thereby enhancing the algorithm’s statistical robustness against harmonic distortion and heavy-tailed non-Gaussian impulse noise.

Additionally, significant progress has been made in high-precision iterative methods and theoretical analysis. Zhe Rao et al. proposed a secondary correction method (RP method) that combines amplitude ratio and segmented phase difference, where the former isolates the main lobe and the latter enables ultra-fine tuning [12]. Chen, K. et al. adopted a hierarchical processing strategy to propose a third-order frequency offset estimation method [13], which is particularly effective for tracking dynamic signals with significant Doppler rates, such as mechanical vibrations. Z. Chen et al. introduced an adaptive tracking algorithm based on an improved Kalman filter [14], facilitating the continuous smoothing of frequency estimates over time.

Liao et al. investigated the underlying mathematical derivation of frequency offset estimation based on all-phase FFT (apFFT) dual-spectral-line interpolation [15]. Owing to the inherent zero phase difference at the main spectral line in apFFT, phase-based interpolation can be effectively applied. Furthermore, they proved that the vast majority of interpolation estimators possess analytical solutions, thereby introducing a completely new set of mathematically unbiased estimators. To tackle the computational complexity of iterative algorithms, Lu et al. proposed an Iterative Interpolated DFT (IpDFT) method that dynamically reconstructs and subtracts the dominant spectral leakage [16], successfully unmasking weak, closely spaced interference components. Wei M. introduced a binary search mechanism (Ds-IpDTFT) to replace complex polynomial root-finding, relieving the reliance on hardware-unfriendly operations [17]. Yang et al. utilized Semidefinite Relaxation (SDR) technology to relax the non-convex cost function of MLE [18], providing reliable global initial values that prevent the Gauss–Newton iteration from becoming trapped in local minima. Liao et al. proved that the interpolated estimator admits an analytical solution [19], thereby introducing an unbiased estimator.

Although significant progress have been made to the development of frequency estimation algorithms, the design of the efficient hardware implementation platforms is also crucial for their practical deployment. Wang et al. pioneered its deployment on FPGAs by designing a highly pipelined architecture that optimizes the utilization of dedicated DSP slices [1]. Ye et al. detailed the hardware implementation of the M-Rife algorithm on FPGAs and successfully applied it to FMCW LiDAR ranging [20], their work verified that RIFE-class algorithms could operate at high sampling rates with minimal Look-Up Table (LUT) and Flip-Flop (FF) resource consumption, demonstrating their feasibility for real-time hardware implementation. For active anti-jamming filter design, Same et al. proposed using simplified Welch power spectrum estimation combined with a chained Infinite Impulse Response (IIR) notch filter structure [21], which facilitates the adaptive tracking and suppression of multiple narrowband interference tones with extremely steep roll-off characteristics. Furthermore, Gomes et al. proposed a software-defined antenna array platform based on the heterogeneous Zynq UltraScale+ RFSoC [22], utilizing the parallel processing capabilities of the Programmable Logic (PL) for high-speed computation (e.g., beamforming and filtering) while placing complex control logic and dynamic weight updates on the Processing System (PS) side. This heterogeneous hardware–software co-design paradigm enhances system flexibility, resource efficiency, and real-time response speed.

Based on the above discussion, to address the inherent conflict between high-precision frequency estimation and microsecond-level real-time responsiveness, a novel interference mitigation scheme based on a Newton-iteration-enhanced three-spectral-line RIFE method and a heterogeneous hardware architecture is proposed. The core contributions of this paper are explicitly summarized as follows:

(1) A Cascaded High-Precision Frequency Estimation Algorithm: We propose a novel Newton-iteration-enhanced three-spectral-line RIFE algorithm. By utilizing the three-spectral-line RIFE for coarse estimation and Newton iteration for fine refinement, this cascaded architecture effectively overcomes the limitations of traditional iterative algorithms, including slow convergence and high sensitivity to initial values. Furthermore, it achieves near-CRLB high-precision frequency estimates with a significantly reduced number of FFT points, making it exceptionally well-suited for tracking highly dynamic sweep-frequency interference.

(2) A Heterogeneous “Fast-Slow Loop Separation” Hardware Architecture: To address the performance degradation and excessive computational loop delays inherent in pure software-based SDR platforms, a tunable, low-latency hardware architecture based on joint PS-PL (Processing System–Programmable Logic) co-design is developed. Specifically, to ensure a microsecond-level rapid response to incoming interference signals, the critical “fast loop”—encompassing interference detection, coarse frequency estimation, and real-time notch filtering—is deployed entirely within the PL (FPGA) fabric. Simultaneously, the PS (ARM Cortex) manages a supervisory “slow loop” responsible for Newton iterative refinement, high-level system control, and the precise calibration of filter coefficients.

(3) An Effective Trade-off Among Accuracy, Latency, and Resource Efficiency: The proposed software-hardware co-design strategy fundamentally breaks the latency bottleneck of traditional high-precision iterative algorithms. By decoupling the ultimate estimation accuracy from the physical FFT size, the proposed platform drastically reduces hardware resource consumption (e.g., BRAM utilization) while significantly extending the overall anti-jamming margin of the GNSS receiver under severe dynamic conditions.

Table 1 summarizes the advantages and disadvantages of different algorithms in terms of estimation accuracy, computational complexity, and response speed.

The remainder of this paper is organized as follows: Section 2 establishes the theoretical framework, detailing the principles of digital notch filters and the rigorous mathematical derivation of the proposed Newton-refined RIFE algorithm. Section 3 describes the hardware implementation, focusing on the joint PS–PL scheduling mechanism and the heterogeneous design of the anti-jamming processor. Section 4 presents the experimental validation. The performance of the proposed algorithm is comprehensively evaluated against conventional methods under both fixed-frequency and sweep-frequency interference scenarios, followed by a closed-loop GNSS positioning test. Finally, Section 5 concludes the paper by summarizing the key contributions and outlining directions for future research.

## 2. Methods

**Figure 2:**
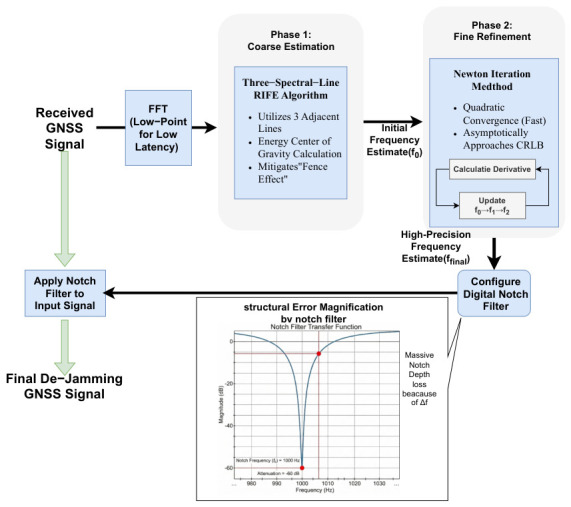
Overview of the proposed methodology.

In the case of a sweep-frequency or other similar jamming signal, minor frequency misalignment between the notch null and the interference center frequency can result in undesired attenuation of the GNSS signal, necessitating the real-time precise estimation of the interference center frequency. The proposed high-precision, real-time anti-jamming scheme, based on a Newton-iteration-enhanced three-spectral-line RIFE algorithm, is designed to resolve the inherent conflict between ultra-high-frequency estimation accuracy and microsecond-level hardware responsiveness in GNSS receivers. This is particularly critical when dealing with highly dynamic interference, such as sweep-frequency jamming. The methodology adopts a hierarchical processing architecture. Initially, an improved three-spectral-line RIFE algorithm is employed to execute a fast coarse estimation by calculating the approximate frequency center within the main lobe. This step effectively mitigates the FFT picket-fence effect while introducing minimal computational overhead. Subsequently, a fine refinement stage is introduced based on the Newton iteration method. By exploiting the intrinsic properties of the Fourier transform, this stage achieves quadratic convergence, allowing the estimation accuracy to approach the Cramér–Rao lower bound (CRLB) within a limited number of iterations. In summary, the proposed methodology, as shown in Figure 2, can improve the performance the notch filter from two aspects, including the accuracy and real-time capability.

### 2.1. Impact of Estimation Error on Anti-Jamming Performance

In conventional anti-jamming architectures, digital notch filtering is widely adopted as the primary technique for suppressing narrowband interference. A notch filter is specifically designed to attenuate signals at targeted frequencies, exhibiting deep rejection at a single narrowband frequency while maintaining an extremely narrow stopband, thereby minimizing distortion to adjacent useful signal components. Such filters can be implemented using either Finite Impulse Response (FIR) or Infinite Impulse Response (IIR) structures. In this work, a single-sided IIR notch filter is employed for interference suppression.

The operating principle of an IIR notch filter is based on the placement of conjugate zeros and poles in the z-plane. To achieve strong attenuation at the interference frequency, the zeros are typically located on the unit circle to generate a deep spectral null. Correspondingly, the poles are placed slightly inside the unit circle at the same angular frequency, ensuring system stability while preserving a narrow stopband. The general transfer function of the filter can be expressed as follows:

(1) $$H\left(z\right)=\frac{1-e^{j\omega_{0}}z^{-1}}{1-pe^{j\omega_{0}}z^{-1}}$$

where $z^{-1}$ denotes the unit-delay operator in the Z-domain, and $\omega_{0}$ represents the notch angular frequency (in radians per sample), which is related to the physical frequency $f_{0}$ (in Hz) and the sampling frequency $f_{s}$ (in Hz) by $\omega_{0}=2\pi f_{0}/f_{s}$. The parameter *p* is the pole radius satisfying 0<p<1, which controls the bandwidth and selectivity of the notch: as *p* approaches unity, the pole moves closer to the unit circle, resulting in a narrower notch and improved frequency selectivity. The constraint |p|<1 ensures the stability of the system.

When the input signal frequency coincides with $\omega_{0}$, the numerator of the transfer function becomes zero while the denominator remains nonzero, thereby producing a transmission zero that effectively suppresses interference at the notch frequency. For frequencies away from $\omega_{0}$, the pole radius being close to unity ensures that the numerator and denominator remain nearly equal in magnitude. Consequently, the magnitude response approaches unity, resulting in negligible attenuation of signal components outside the stopband and thus preserving the integrity of the desired signal.

By applying the inverse Z-transform, the transfer function can be equivalently expressed in the time domain as the following difference equation:

(2) $$y\left[n\right]-pe^{j\omega_{0}}y\left[n-1\right]=x\left[n\right]-e^{j\omega_{0}}x\left[n-1\right]$$

Given that both the input and output of the notch filter are complex baseband signals, they can be defined as x[n]=I[n]+jQ[n] and $y\left[n\right]=I_{out}\left[n\right]+jQ_{out}\left[n\right]$. To minimize computational complexity for hardware implementation, trigonometric coefficients are introduced as $c_{r}=\cos\omega_{0}$ and $c_{i}=\sin\omega_{0}$. By substituting these defined parameters into the generalized difference equation, the resulting expanded expression is obtained, which is specifically optimized for hardware instantiation within the FPGA fabric.

(3) $$\begin{array}{cc} I_{out}\left[n\right] & =I\left[n\right]-\left(c_{r}I\left[n-1\right]-c_{i}Q\left[n-1\right]\right)+p\left(c_{r}I_{out}\left[n-1\right]-c_{i}Q_{out}\left[n-1\right]\right)\\ Q_{out}\left[n\right] & =Q\left[n\right]-\left(c_{r}Q\left[n-1\right]+c_{i}I\left[n-1\right]\right)+p\left(c_{r}Q_{out}\left[n-1\right]+c_{i}I_{out}\left[n-1\right]\right) \end{array}$$

The preceding analysis indicates that as the pole radius *p* approaches unity, the magnitude response of the filter asymptotically approaches 1. Consequently, signal components outside the notch frequency experience negligible attenuation, resulting in an extremely narrow stopband. By evaluating the frequency response under the standard −3 dB half-power bandwidth criterion, the analytical expression for the notch bandwidth can be derived as follows:

(4) $$\frac{1}{\sqrt{2}}=\left|\frac{1-e^{j\frac{B_{rad}}{2}}}{1-pe^{j\frac{B_{rad}}{2}}}\right|\approx \left|\frac{j\frac{B_{rad}}{2}}{\left(j\frac{B_{rad}}{2}-\ln\left(p\right)\right)}\right|$$

Taking the modulus of both sides yields the half-power bandwidth $B_{rad}\approx -2\ln\left(p\right)$. This analytical expression quantitatively determines the bandwidth size. However, in practical anti-jamming applications, digital notch filters typically do not impose strict bandwidth constraints. Instead, the primary objective is to effectively suppress the interference while ensuring minimal distortion to adjacent useful signal components. For the hardware implementations detailed in this study, the pole radius is empirically set to p=0.995.

Unlike an ideal notch filter that achieves infinite attenuation at the interfering frequency, a practical digital notch filter exhibits a sharp attenuation dip in the vicinity of its center frequency. This sharp transition implies that the notch depth degrades rapidly as the frequency deviates from the exact null point. Consequently, this inherent sensitivity necessitates highly accurate frequency estimation. Even small estimation errors are significantly amplified by the steep frequency response, leading to a notable degradation in the effective notch depth. To quantitatively analyze the impact of frequency estimation errors, the actual input frequency is expressed as $z=e^{j(\omega_{0}+\Delta \omega )}$, where Δω denotes the frequency estimation error. Substituting this expression into the system model, the transfer function of the notch filter can be formulated as follows:

(5) $$H\left(\omega \right)=\frac{1-e^{j\omega_{0}}\cdot e^{-j\omega_{0}-\Delta \omega }}{1-pe^{j\omega_{0}}\cdot e^{-j\omega_{0}-\Delta \omega }}=\frac{1-e^{-\Delta \omega }}{1-pe^{-\Delta \omega }}$$

To derive the magnitude of the frequency response for subsequent gain evaluation, the squared moduli of both the numerator and the denominator must be calculated. By expanding and simplifying these terms, the squared moduli can be expressed as follows:

(6) $$\begin{array}{cc} |1-e^{-j\Delta \omega }{|}^{2} & ={\left|1-\left(\cos\Delta \omega -j\sin\Delta \omega \right)\right|}^{2}=2-2\cos\Delta \omega =4\sin^{2}\frac{\Delta \omega }{2} \end{array}$$

(7) $$\begin{array}{cc} |1-pe^{-j\Delta \omega }{|}^{2} & ={\left|1-p\left(\cos\Delta \omega -j\sin\Delta \omega \right)\right|}^{2}=1-2p\cos\Delta \omega +p^{2} \end{array}$$

Thus, the final amplitude response function is obtained as

(8) $$\left|H\left(\Delta \omega \right)\right|=\sqrt{\frac{2(1-\cos\Delta \omega )}{1-2p\cos\Delta \omega +p^{2}}}$$

To further simplify the resulting expression, the frequency estimation error Δω is assumed to be sufficiently small. By applying the small-value approximation, the equation can be systematically reduced to

(9) $$\left|H\left(\Delta \omega \right)\right|\approx \sqrt{\frac{2(1-\left(1-\frac{\Delta \omega^{2}}{2}\right))}{1-2p\left(1-\frac{\Delta \omega^{2}}{2}\right)+p^{2}}}=\sqrt{\frac{\Delta \omega^{2}}{{\left(1-p\right)}^{2}+p\Delta \omega^{2}}}=\frac{\left|\Delta \omega \right|}{\sqrt{{\left(1-p\right)}^{2}+p\Delta \omega^{2}}}$$

In practical frequency estimation scenarios, the maximum estimation error generally does not exceed 2 kHz and is typically within several hundred hertz. Considering that the actual sampling rate in this experiment is 30.72 MHz and the empirical pole radius is set to p=0.995, the maximum estimation error of 2 kHz corresponds to a normalized angular frequency deviation of approximately 0.0004rad. Therefore, the term $p\Delta \omega^{2}$ in the formula can be regarded as negligible compared with ${\left(1-p\right)}^{2}$. Consequently, in practical engineering applications, the term $p\Delta \omega^{2}$ can be approximated as a higher-order infinitesimal quantity. Therefore, higher-order error terms in the denominator can be neglected, and the magnitude of the transfer function can be simplified as follows:

(10) $$\left|H\left(\Delta \omega \right)\right|=\frac{\left|\Delta \omega \right|}{1-p}$$

The equation above explicitly demonstrates that in the immediate vicinity of the notch frequency, the magnitude response of the filter is directly proportional to the frequency estimation error. Crucially, due to the denominator effect, this initial error is mathematically amplified by a factor of $\frac{1}{1-p}$. Given the empirical configuration of p=0.995 adopted in this paper, the error is drastically magnified by a factor of 200. This analytical relationship underscores that even the most marginal frequency estimation error will degrade the effective notch depth and the overall suppression performance of the filter.

### 2.2. Conventional Frequency Estimation Methods

The RIFE algorithm is an efficient FFT-based approach for sub-bin frequency estimation. Its fundamental principle exploits the mathematical characteristics of the FFT main lobe, particularly the sinc-shaped amplitude envelope, by interpolating the amplitude ratios of adjacent spectral lines. This mechanism enables accurate extraction of the fractional frequency offset, thereby overcoming the inherent resolution limitations of conventional FFT-based methods.

However, the conventional RIFE algorithm relies solely on the primary and secondary spectral lines. When the true signal frequency is close to an integer frequency bin, the amplitude of the secondary spectral component becomes significantly attenuated. Under low signal-to-noise ratio (SNR) conditions, this weak component is highly susceptible to noise contamination, which may lead to incorrect estimation of the frequency offset direction. To address this limitation and further improve estimation accuracy, an enhanced three-spectral-line RIFE algorithm is proposed. By incorporating the three dominant spectral components around the peak, the method introduces additional spectral information to suppress noise-induced variance, thereby providing a more robust and accurate initial frequency estimate for subsequent Newton iteration.

In a standard *N*-point FFT, the frequency resolution is fundamentally limited by the number of observed samples, yielding a bin spacing of $F_{s}/N$. In contrast, the RIFE algorithm overcomes this limitation by analytically estimating the fractional frequency offset within a discrete Fourier bin, thereby achieving sub-bin frequency resolution. For a discrete-time single-tone signal, the time-domain model is given by

(11) $$x\left[n\right]=A\cdot \cos\left(2\pi f_{0}\cdot \frac{n}{F_{s}}+\phi \right),n=0,1,2,\ldots ,N-1$$

where *A* denotes the signal amplitude, $f_{0}$ is the sinusoidal frequency, $F_{s}$ is the sampling frequency, ϕ represents the initial phase, and *N* is the total number of samples. By applying Euler’s formula, the cosine signal can be decomposed into a sum of two complex exponentials as

(12) $$x\left[n\right]=\frac{A}{2}\left[e^{j\left(2\pi \frac{f_{0}}{F_{s}}n+\phi \right)}+e^{-j\left(2\pi \frac{f_{0}}{F_{s}}n+\phi \right)}\right]$$

After expansion, the DFT transformation is performed to obtain the frequency-domain components of both positive and negative frequencies:

(13) $$X\left[k\right]=\frac{A}{2}\left[X_{1}\left[k\right]+X_{2}\left[k\right]\right]=e^{j\phi }\cdot \sum_{n=0}^{N-1}e^{j2\pi n\left(\frac{f_{0}}{F_{s}}-\frac{k}{N}\right)}+e^{-j\phi }\cdot \sum_{n=0}^{N-1}e^{-j2\pi n\left(\frac{f_{0}}{F_{s}}-\frac{k}{N}\right)}$$

where X[k] denotes the *k*-th DFT coefficient, k=0,1,…,N−1. By summing their geometric series, we can obtain:

(14) $$\left|X\left[k\right]\right|=\left|\frac{\sin\left(\pi \left(\frac{Nf_{0}}{F_{s}}-k\right)\right)}{\sin\left(\frac{\pi }{N}\left(\frac{Nf_{0}}{F_{s}}-k\right)\right)}+\frac{\sin\left(\pi \left(\frac{Nf_{0}}{F_{s}}+k\right)\right)}{\sin\left(\frac{\pi }{N}\left(\frac{Nf_{0}}{F_{s}}+k\right)\right)}\right|$$

Given that the primary objective is to evaluate the spectral peak, the analysis focuses on the local neighborhood of the main lobe, where the spectral index is given by $k\approx \frac{f_{0}}{F_{s}}\cdot N=k_{0}$. Consequently, the conjugate term $X_{2}\left[k\right]$ approaches zero, i.e., $X_{2}\left[k\right]\approx \frac{\sin(2k_{0}\pi )}{\sin\left(\frac{\pi }{N}\left(\frac{Nf_{0}}{F_{s}}+k\right)\right)}\approx 0$. By neglecting this highly attenuated component, the magnitude of the spectral response simplifies to:

(15) $$\left|X\left[k\right]\right|\approx \left|\frac{\sin\left(\pi \left(\frac{Nf_{0}}{F_{s}}-k\right)\right)}{\sin\left(\frac{\pi }{N}\left(\frac{Nf_{0}}{F_{s}}-k\right)\right)}\right|$$

To rigorously quantify the influence of the fractional frequency offset on the main lobe amplitude, the normalized frequency deviation $\Delta =\frac{Nf_{0}}{F_{s}}-k_{0}$ and the relative spectral bin index $m=k-k_{0}$ are introduced. Substituting these defined variables into Equation (15) yields:

(16) $$|X\left[k_{0}+m\right]|\approx \left|\frac{A}{2}\frac{\sin(\pi (\Delta -m))}{\sin\left(\frac{\pi }{N}\left(\Delta -m\right)\right)}\right|$$

In practical implementations, to ensure sufficient spectral resolution, the FFT size *N* is typically configured to be substantially large. This leads to a sufficiently small argument $\frac{\pi (\Delta -m)}{N}$. By applying the small-angle approximation to the denominator, the expression is further systematically reduced to:

(17) $$|X\left(k_{0}+m\right)|\approx \frac{A\cdot N}{2\pi }\left|\frac{\sin(\pi (\Delta -m))}{\Delta -m}\right|=\frac{A\cdot N}{2}\;\mathrm{sinc}\left(\Delta -m\right)$$

As indicated by the preceding formula, the Discrete Fourier Transform (DFT) of a single-tone signal can be accurately approximated by a Sinc function in the vicinity of its main spectral peak. Furthermore, the amplitude within this main lobe is strictly a function of the fractional frequency offset and the relative bin index. Consequently, for a given relative bin index, the precise frequency offset can be analytically deduced by evaluating the amplitudes of adjacent spectral bins.

A Taylor expansion of Equation (17) at $k_{0}$ yields:

(18) $$|X\left(k_{0}\right)|\approx \frac{A\cdot N}{2}\left(1-\frac{\pi^{2}{\left(k_{0}\right)}^{2}}{6}+O\left({\left(k_{0}\right)}^{4}\right)\right)$$

The expanded equation explicitly reveals that the main lobe of the spectral peak can be effectively modeled utilizing a quadratic fitting function. Notably, the absence of a third-order error term inherently guarantees a high fitting precision. Let $Y_{-1},Y_{0},Y_{+1}$ denote the magnitude values of the three adjacent spectral bins centered directly around the peak obtained from the FFT. The fractional frequency offset can thus be calculated as follows:

(19) $$\Delta =\frac{Y_{-1}-Y_{+1}}{2(Y_{-1}-2Y_{0}+Y_{+1})}$$

### 2.3. Improved Spectral Estimation Method

Although the enhanced three-spectral-line RIFE algorithm effectively mitigates the picket-fence effect inherent in the FFT and maintains robust performance under low signal-to-noise ratio (SNR) conditions, its estimation variance still does not reach the theoretical Cramér–Rao lower bound (CRLB). To further reduce the residual frequency bias and achieve near-optimal estimation accuracy, this paper proposes a cascaded frequency estimation scheme that integrates the initial three-spectral-line RIFE estimate with a subsequent Newton iterative refinement process.

Let x[n] denote a discrete-time observation signal. Its corresponding Discrete-Time Fourier Transform (DTFT) is mathematically defined as follows:

(20) $$X\left(\omega \right)=\sum_{n=0}^{N-1}x\left[n\right]\cdot e^{-j\omega n}$$

where ω is the normalized angular frequency. The maximum frequency of power spectral density (PSD) is needed to estimate the frequency.

Define the objective function J(ω) as the square of the signal’s spectral modulus:

(21) $$J\left(\omega \right)={\left|X\left(\omega \right)\right|}^{2}=X\left(\omega \right)\cdot X^{*}\left(\omega \right)$$

At this stage, the high-precision frequency estimation problem is rigorously formulated as an unconstrained optimization problem seeking the global extremum of an objective function. According to the first-order optimality condition, this optimization can be further reduced to determining the roots of the derivative equation $J^{\prime }\left(\omega \right)=0$. To avoid the cumbersome direct differentiation of complex analytical expressions in the frequency domain, this paper leverages the analytical derivative property of the Discrete-Time Fourier Transform (DTFT). This elegant mathematical property seamlessly converts the complex frequency-domain differentiation into a straightforward time-domain weighting operation. Based on the definition of X(ω), its first and second-order derivatives are analytically derived as follows:

(22) $$\begin{array}{cc} X^{\prime }\left(\omega \right) & =\frac{d}{d\omega }\sum_{n=0}^{N-1}x\left[n\right]\cdot e^{-j\omega n}=\sum_{n=0}^{N-1}\left(-jn\right)\cdot x\left[n\right]\cdot e^{-j\omega n}=DTFT\left\{-jn\cdot x\left[n\right]\right\} \end{array}$$

(23) $$\begin{array}{cc} X^{\prime \prime }\left(\omega \right) & =\frac{d}{d\omega }X^{\prime }\left(\omega \right)=\sum_{n=0}^{N-1}{\left(-jn\right)}^{2}\cdot x\left[n\right]\cdot e^{-j\omega n}=DTFT\left\{-n^{2}\cdot x\left[n\right]\right\} \end{array}$$

The aforementioned derivations demonstrate that computing the first derivative in the frequency domain is mathematically equivalent to performing a DTFT on the original time-domain signal multiplied by a linear window. Similarly, the second derivative corresponds to the DTFT of the time-domain signal subjected to a parabolic window.

This algorithmic simplification completely bypasses computationally expensive trigonometric differentiations. Furthermore, it is highly suitable for hardware implementation, enabling efficient utilization of FPGA resources and achieving significant acceleration in processing speed.

Given an objective function J(ω), its first and second derivatives can thus be explicitly formulated in terms of X(ω) and its derivatives:

(24) J′(ω)=ddω[X(ω)·X∗(ω)]=X′(ω)·X∗(ω)+X(ω)[X′(ω)]∗=2Re{X′(ω)X∗(ω)}

The formula Re{} represents the real part of the result. Similarly, we can obtain the second derivative of J(ω):

(25) J′′(ω)=ddωJ′(ω)=2Re{X′′(ω)·X∗(ω)+X′(ω)·[X′(ω)]∗}=2Re{X′′(ω)·X∗(ω)}+2|X′(ω)|2

The Newton iteration method fundamentally relies on a Taylor series expansion to linearize the first derivative of the objective function. The generalized iterative update rule is defined as

(26) $$\omega_{k+1}=\omega_{k}-\frac{J^{\prime }\left(\omega_{k}\right)}{J^{\prime \prime }\left(\omega_{k}\right)}$$

where $\omega_{k}$ denotes the frequency estimate at the k-th iteration, and μ represents the iteration step size. To guarantee rapid and stable convergence, the initial seed value is directly provided by the proposed three-spectral-line RIFE algorithm. This high-precision coarse initialization is an absolute prerequisite for unlocking the quadratic convergence characteristic of the Newton method, effectively preventing the optimization process from becoming trapped in local minima. By substituting the previously derived analytical expressions for the objective function’s derivatives into Equation (26), the explicit iteration formula for frequency estimation is formulated as

(27) ωk+1=ωk−μRe{X′(ω)·X∗(ω)}Re{X′′(ω)·X∗(ω)}+|X′(ω)|2

The fundamental frequency resolution of conventional FFT-based estimation algorithms is strictly constrained by the frequency bin width, $\Delta f=\frac{f_{s}}{N}$, which is a direct consequence of the picket-fence effect. To enhance estimation accuracy, traditional approaches are inevitably forced to increase the FFT size N. Furthermore, because most conventional algorithms rely heavily on localized approximation techniques, their estimation performance degrades precipitously if the FFT point count is excessively reduced. Consequently, achieving high precision traditionally mandates a large *N*, which invariably exacerbates data buffering latency and escalates the computational complexity to O(NlogN). In contrast, the proposed cascaded architecture—integrating the Newton-iteration-enhanced three-spectral-line RIFE—fundamentally exploits the local convexity of the objective function. By analytically computing the derivatives to pinpoint the extremum, this iterative scheme guarantees rapid convergence to the true frequency, provided that the initial RIFE estimate falls securely within the main lobe of the actual signal spectrum. Crucially, this innovative design completely decouples the ultimate frequency estimation accuracy from the physical FFT point count. In this proposed architecture, the FFT size merely serves to provide a reliable initial coarse estimate with minimal error, rather than bounding the final theoretical precision. Therefore, a significantly reduced FFT point count can be employed to rapidly acquire a robust initial value. The ultra-high precision, traditionally necessitating massive FFT sizes, is subsequently achieved through Newton iterations characterized by a negligible computational complexity of O(1). This architectural breakthrough dramatically minimizes data buffering requirements and slashes the overall system response latency, rendering it highly optimal for real-time anti-jamming applications.

Having established the algorithmic framework and computational advantages of the proposed cascaded frequency estimation scheme, it is necessary to further examine its convergence properties. It is well known that the performance of the Newton–Raphson method critically depends on the quality of the initial estimate; poor initialization may lead to divergence or convergence to undesired local optima. The proposed approach assumes that the enhanced three-spectral-line RIFE algorithm provides an initial frequency estimate located within the convex region of the objective function around the main lobe. Under this condition, the Newton iteration can reliably achieve stable convergence. To validate this assumption and ensure the theoretical soundness of the overall framework, the following section presents a rigorous convergence analysis. It is analytically shown that, given the RIFE-based initialization, the iterative refinement process converges to the global optimum.

For the single-tone signal input environment, according to Formula (16), we can obtain:

(28) $$J\left(\omega \right)={\left|X\left(\omega \right)\right|}^{2}=\frac{A^{2}}{4}{\left(\frac{\sin\frac{N(\omega -\omega_{0})}{2}}{\sin\frac{\omega -\omega_{0}}{2}}\right)}^{2}$$

Given the error of the frequency estimate at the k-th iteration as $\epsilon_{k}=\omega_{k}-\omega_{0}$, we can obtain:

(29) $$\epsilon_{k+1}=\epsilon_{k}-\frac{J^{\prime }\left(\omega_{k}\right)}{J^{\prime \prime }\left(\omega_{k}\right)}$$

This paper employs the three-spectral-line RIFE algorithm to provide initial values, which means that the initial error is already relatively small. Since $\omega_{0}$ represents the true frequency corresponding to the spectral peak of the interference, it satisfies the first-order optimality condition $J^{\prime }\left(\omega_{0}\right)=0$. By performing a Taylor expansion on the function J(ω), we can obtain:

(30) $$J^{\prime }\left(\omega_{k}\right)=J^{\prime }\left(\omega_{0}\right)+J^{\prime \prime }\left(\omega_{0}\right)\left(\omega_{k}-\omega_{0}\right)+\frac{1}{2}J^{\prime \prime \prime }\left(\xi \right){\left(\omega_{k}-\omega_{0}\right)}^{2}$$

Similarly, we can get:

(31) $$\epsilon_{k+1}\approx \frac{\epsilon_{k}\left[J^{\prime \prime }\left(\omega_{0}\right)+J^{\prime \prime \prime }\left(\omega_{0}\right)\epsilon_{k}\right]-\left[J^{\prime \prime }\left(\omega_{0}\right)\epsilon_{k}+\frac{1}{2}J^{\prime \prime \prime }\left(\omega_{0}\right)\epsilon_{k}^{2}\right]}{J^{\prime \prime }\left(\omega_{0}\right)+J^{\prime \prime \prime }\left(\omega_{0}\right)\epsilon_{k}}$$

It should be noted that although the initial value $\omega_{0}$ is obtained through the preceding coarse frequency alignment stage, which generally prevents the iterative method from converging to a local optimum, it is still necessary to verify that $J^{\prime \prime }\left(\omega_{0}\right)<0$ to ensure that the final convergence point corresponds to a maximum rather than a local minimum. Since $\epsilon_{k}$ can be regarded as a small quantity, terms containing higher-order small quantities can be neglected, and the result can be obtained:

(32) $$\epsilon_{k+1}\approx \frac{J^{\prime \prime \prime }\left(\omega_{0}\right)\epsilon_{k}^{2}-\frac{1}{2}J^{\prime \prime \prime }\left(\omega_{0}\right)\epsilon_{k}^{2}}{J^{\prime \prime }\left(\omega_{0}\right)+J^{\prime \prime \prime }\left(\omega_{0}\right)\epsilon_{k}}=\frac{\frac{1}{2}J^{\prime \prime \prime }\left(\omega_{0}\right)\epsilon_{k}^{2}}{J^{\prime \prime }\left(\omega_{0}\right)+J^{\prime \prime \prime }\left(\omega_{0}\right)\epsilon_{k}}\approx \frac{J^{\prime \prime \prime }\left(\omega_{0}\right)}{2J^{\prime \prime }\left(\omega_{0}\right)}\epsilon_{k}^{2}$$

which means

(33) $$\lim_{k\to \infty }\frac{|\epsilon_{k+1}|}{|\epsilon_{k}{|}^{2}}=C$$

Consequently, this analytical property indicates that the proposed algorithm exhibits quadratic convergence, enabling rapid convergence to the true frequency within a small number of iterations. Moreover, since the derivation does not rely on heuristic approximations, the resulting estimator is theoretically unbiased and asymptotically approaches the Cramér–Rao Lower Bound (CRLB).

### 2.4. Algorithm Performance Simulation Experiments

Although the preceding analysis establishes the theoretical convergence and asymptotic optimality of the proposed algorithm, empirical validation is necessary to assess its practical robustness. Therefore, comprehensive numerical simulations are conducted to evaluate the frequency estimation performance under varying Signal-to-Noise Ratio (SNR) conditions.

To further quantify the performance of the proposed cascaded algorithm, simulations are carried out using the MATLAB 2022 platform. A single-tone signal model corrupted by Additive White Gaussian Noise (AWGN) is considered, with the FFT size set to N=4096. To ensure statistical reliability, 5000 independent Monte Carlo trials are performed. In each trial, the true signal frequency and initial phase are randomly generated according to a uniform distribution over predefined intervals. The performance is evaluated using the Root Mean Square Error (RMSE) and the Mean Absolute Error (MAE), while the theoretical performance bound is given by the Cramér–Rao Lower Bound (CRLB).

**Figure 3:**
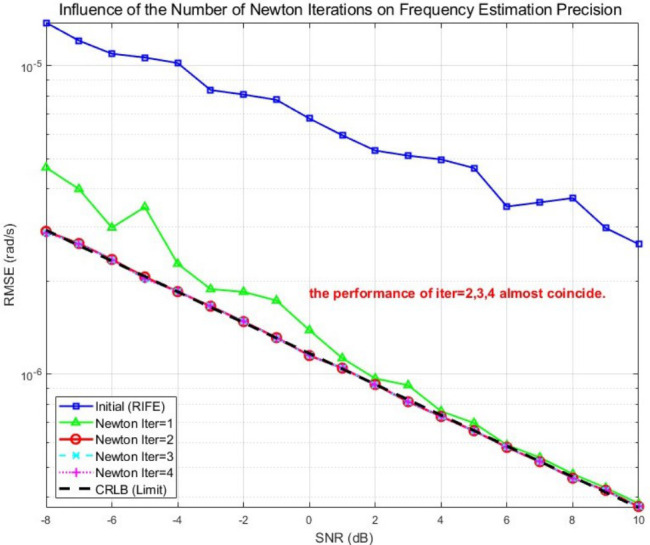
Estimation of the accuracy of Newton’s iteration method with different iteration times.

Within the context of the Newton iteration method, the requisite number of iterations constitutes a critical parameter, as it directly dictates both the computational complexity and the real-time execution capabilities of the hardware. To systematically assess the impact of this parameter, Figure 3 illustrates the relationship between the algorithm’s estimation precision and the iteration count.

Figure 3 illustrates the evolution of estimation accuracy with respect to the iteration count. After the first iteration, the accuracy improves significantly compared to the initial estimate but remains slightly above the CRLB, indicating incomplete convergence. When the iteration count reaches k=2, the RMSE decreases sharply and nearly coincides with the theoretical CRLB. This result empirically validates the convergence analysis of the Newton method and confirms its quadratic convergence property, effectively eliminating residual frequency bias. Further increasing the iteration count beyond two yields only marginal improvement, with the RMSE curves nearly overlapping.

From a hardware implementation perspective, each additional iteration incurs increased FPGA resource consumption and processing latency. Therefore, to achieve an optimal trade-off between estimation accuracy and hardware efficiency (i.e., low latency and reduced resource utilization), a two-iteration configuration is adopted in the final design.

**Figure 4:**
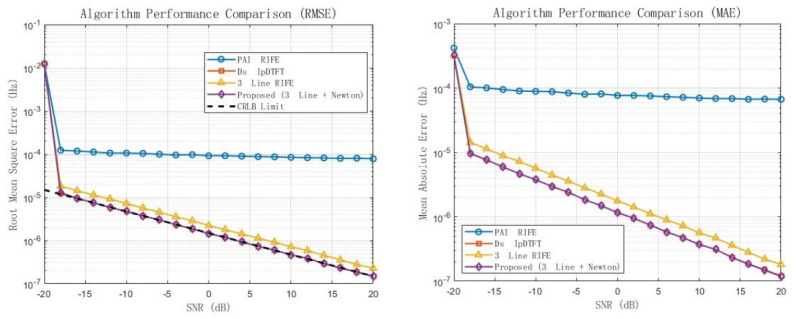
Performance comparison between different algorithms.

Based on the optimized iteration-number configuration described above, comprehensive comparative experiments were conducted in this section to thoroughly evaluate the estimation performance and superiority of the proposed algorithm. The experiments introduced PAI-RIFE and Ds-IpDTFT as baseline methods, which respectively represent advanced closed-form interpolation-based estimation algorithms and high-precision iterative search algorithms. This provides scientifically grounded references for comparative validation from different methodological perspectives. Figure 4 presents the corresponding comparison results.

Simulation results demonstrate that the proposed cascaded algorithm, which combines the three-spectral-line RIFE method with Newton iteration, significantly outperforms both PAI-RIFE and the standalone three-spectral-line RIFE algorithm in terms of root mean square error (RMSE) and mean absolute error (MAE). Moreover, with only a small number of iterations, the proposed method achieves an optimal estimation accuracy comparable to that of the state-of-the-art Ds-IpDTFT algorithm. It can also be observed that the improved three-spectral-line RIFE algorithm, benefiting from the incorporation of richer adjacent spectral-line information, exhibits superior performance over PAI-RIFE under the experimental conditions considered in this study.

Under the extremely low signal-to-noise ratio (SNR) condition of −20dB, severe deviations in the initial peak-search process caused by strong noise lead to significant performance degradation for all evaluated algorithms due to the threshold effect. Nevertheless, such extremely low-SNR environments are rarely encountered in typical anti-jamming tracking scenarios. As the SNR increases, the proposed method rapidly converges and achieves remarkably high estimation accuracy owing to its rigorous DTFT derivative analytical model. Notably, the error curve of the proposed method almost completely overlaps with that of Ds-IpDTFT and closely approaches the theoretical Cramér–Rao lower bound (CRLB). This not only effectively verifies the superiority of the proposed algorithm as a statistically unbiased estimator, but also further highlights its significant advantage in low-complexity hardware implementation while maintaining the same limiting estimation accuracy.

**Figure 5:**
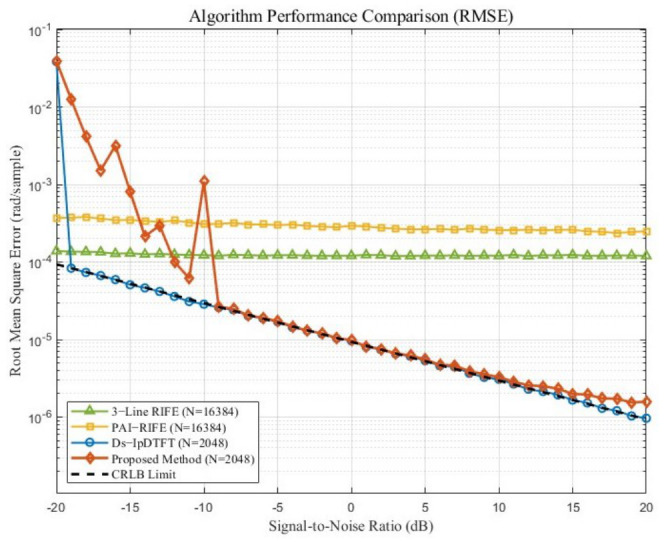
Comparison of algorithm performance under different FFT points.

To further investigate the algorithm’s robustness under restricted FFT lengths, Figure 5 presents a comparative analysis between the conventional algorithm and the proposed three-spectral-line RIFE-Newton scheme. Specifically, it highlights their respective performance when the FFT point count is reduced by a factor of eight:

The comparative analysis presented in Figure 5 demonstrates that, despite the substantial reduction in FFT size, the proposed Newton-iteration-enhanced three-spectral-line RIFE algorithm exhibits excellent performance within the medium-to-high signal-to-noise ratio region (SNR≥−8dB). Its estimation accuracy not only consistently surpasses that of the conventional 16,384-point PAI-RIFE and three-spectral-line RIFE algorithms, but also closely approaches the Cramér–Rao lower bound, achieving a level of optimal performance comparable to the advanced Ds-IpDTFT algorithm under the same FFT size configuration.

It is worth noting that, under extremely high-SNR conditions, the absolute estimation accuracy of the proposed method is slightly inferior to that of the Ds-IpDTFT algorithm due to the limitation imposed by the fixed two-iteration configuration. However, Ds-IpDTFT generally requires more than 15 intensive search iterations to achieve convergence, whereas the proposed method completes the fine estimation process using only a very small number of Newton iterations (two iterations in this work). This engineering strategy, which sacrifices only a marginal amount of estimation accuracy in exchange for an orders-of-magnitude reduction in computational complexity, substantially decreases the processing latency in practical hardware implementations, thereby ensuring microsecond-level dynamic response capability when confronting high-speed sweep jamming signals.

On the other hand, within the extremely low-SNR range from −20dB to −10dB, the proposed method exhibits severe performance fluctuations and localized degradation. This phenomenon is primarily attributed to the intrinsic limitation of small-size FFT processing. Under strong noise interference, if the peak position obtained during the coarse estimation stage deviates significantly from the true frequency location, the subsequent Newton iteration cannot be initialized within the valid quadratic convergence region, ultimately leading to iterative divergence. Consequently, under such harsh low-SNR conditions, the instantaneous estimation error of the proposed method becomes significantly larger than that of the conventional 16,384-point algorithms, which maintain relatively stable performance owing to their higher processing gain associated with large-size FFT operations.

Nevertheless, it must be emphasized that, in practical electronic warfare and anti-jamming environments, the power of suppressive jamming signals is typically far greater than that of the target signal and the background noise. As a result, frequency estimation tasks for jamming sources are generally performed under medium-to-high SNR conditions, and such extremely adverse low-SNR scenarios are rarely encountered in real-world engineering applications. Therefore, this localized theoretical performance limitation does not diminish the core practical value of the proposed method in realistic application scenarios.

## 3. Hardware Design

### 3.1. Design of RF Transceiver Circuit

**Figure 6:**
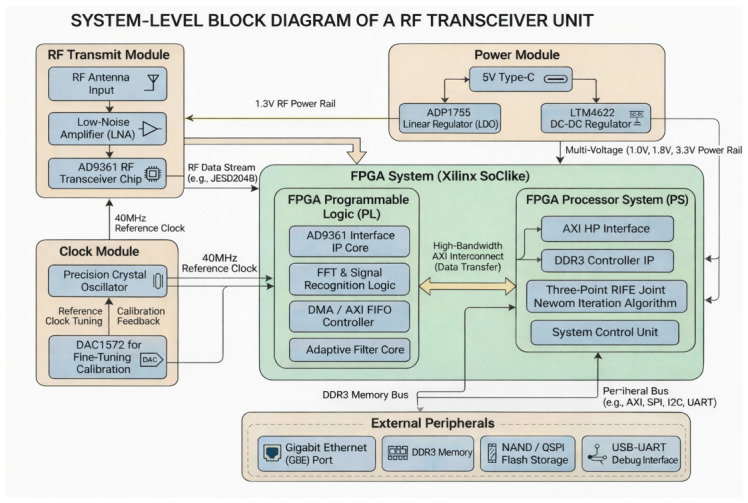
System block diagram.

The RF transceiver constitutes the analog front-end of the system, directly determining the quality of signal acquisition and, consequently, the accuracy of frequency estimation. The proposed system is built upon the AD9361 (Analog Devices, Wilmington, MA, USA), a highly integrated RF agile transceiver with multiple-input multiple-output (MIMO) capability and a wide operating frequency range from 70 MHz to 6 GHz. This architecture enables flexible reconfiguration of key RF parameters, including sampling rate, center frequency, bandwidth, and gain, thereby supporting adaptive single-tone signal reception across multiple frequency bands. Figure 6 shows the overall hardware architecture of the proposed system.

To improve receiver sensitivity, a low-noise amplification stage is integrated within the AD9361 receiving chain using the PGA-102 high-linearity amplifier (Mini-Circuits, Brooklyn, NY, USA). In addition, RF baluns are employed at the signal interfaces to ensure proper single-ended to differential conversion and impedance matching. This design preserves signal integrity while minimizing transmission distortion.

Given the sensitivity of RF circuits to power supply noise, the power management subsystem adopts dual ADP1755 low-dropout (LDO) regulators (Analog Devices, Wilmington, MA, USA) to guarantee stable operation of the AD9361. These LDOs provide a low-noise 1.3 V supply with minimal ripple, stabilizing critical internal blocks such as RF synthesizers, voltage-controlled oscillators (VCOs), and integrated low-noise amplifiers (LNAs). By effectively suppressing power supply ripple-induced interference, the proposed power architecture enhances overall RF front-end stability and reduces residual estimation errors in subsequent frequency processing stages.

### 3.2. Design of Signal Processing Part

At the heart of the system architecture lies the signal processing module, where the majority of computational tasks are executed. The Zynq XC7Z020 SoC (Xilinx, San Jose, CA, USA) was selected for its abundant programmable logic resources, which facilitate the high-speed implementation of FFT pipelines and digital notch filters. This heterogeneous architecture orchestrates a dual-loop strategy: a “fast loop” within the PL handles high-throughput data streams to satisfy stringent real-time requirements, while a “slow loop” within the Processing System manages complex iterative logic to accommodate diverse precision demands across varying operational scenarios.

Within the Programmable Logic, the integration of the AD9361 intellectual property core facilitates the seamless conversion of LVDS streams into 12-bit IQ format. A multi-stage pipelined architecture is employed for the FFT recognition module to ingest and process high-speed IQ data from the RF front-end. This optimized design ensures a rapid frequency response while maintaining peak throughput. Specifically, the module leverages the three-spectral-line Recursive Interpolated FFT algorithm to calculate the high-order derivatives required for the subsequent Newton iterative process. To ensure efficient inter-layer communication, the resulting FFT data is buffered in a FIFO and transferred to the PS via Direct Memory Access over the high-performance AXI-HP interface for final processing.

Within the Processing System, high-performance AXI-HP interfaces and DDR3 memory are integrated to facilitate the rapid transfer and storage of massive data streams generated by the Programmable Logic. Furthermore, the control module of the official AD9361 IP core is incorporated to dynamically configure critical RF parameters of the AD9361 transceiver, such as the center frequency, bandwidth, and gain. Upon receiving the extensive data from the PL, the PS executes secondary data processing and fine-grained estimation, specifically completing the target frequency calculation via the Newton iteration method. Concurrently, the PS adaptively regulates the entire anti-jamming module based on the system’s output, ensuring robust mitigation across diverse interference scenarios.

To support the diverse voltage rails required by the SoC and its peripherals, the power management system utilizes the LTM4622 high-efficiency DC/DC Module regulator (Analog Devices, Wilmington, MA, USA). By delivering dual high-current outputs from a single-chip solution and integrating optimized peripheral circuitry, it ensures high power-conversion efficiency and long-term stability under heavy computational workloads.

### 3.3. Clock Module Design and Peripheral

The precision of frequency estimation is fundamentally constrained by the stability of the system’s reference clock. To ensure a highly stable frequency output, this design incorporates a Voltage-Controlled Temperature-Compensated Crystal Oscillator. Given the system’s high sensitivity to frequency variations, even marginal errors in the reference oscillator can be amplified and compounded through the RF synthesis and up-conversion loops. To mitigate these frequency drifts and maintain synchronization across critical parameters—such as center frequency and bandwidth—the DAC7512 (Texas Instruments, Dallas, TX, USA) is employed for fine-grained voltage control and calibration of the VCTCXO. This feedback mechanism delivers a precise and stabilized 40 MHz reference clock to both the AD9361 RF transceiver and the Zynq FPGA, ensuring optimal system-wide coherence and performance.

To facilitate real-time debugging and telemetry, the system provides a versatile array of communication interfaces. A CP2105 dual-channel USB-to-UART bridge (Silicon Labs, Austin, TX, USA) is integrated to provide two independent serial ports, connecting to the Zynq PS and PL modules respectively for efficient logging and status monitoring. Furthermore, a dedicated Ethernet Physical Layer chip supports standard UDP/TCP protocols, enabling the high-bandwidth streaming of raw IQ samples and frequency offset estimates to a host computer for further offline analysis or real-time visualization.

### 3.4. Hardware Resource Consumption and End-to-End System Latency Analysis

To provide a more comprehensive evaluation of the hardware platform performance, this section presents a detailed analysis of the hardware resource overhead of the proposed heterogeneous anti-jamming architecture implemented on the Zynq-7000 SoC platform, together with a rigorous decomposition of the physical latency introduced by each processing stage of the system. Through quantitative analysis, the core advantage of the small-size FFT design in mitigating highly dynamic sweep jamming is further demonstrated.

Benefiting from the optimization of the proposed algorithm, the FFT data window can be aggressively reduced to 2048 points. This reduction in FFT size substantially alleviates the hardware resource utilization on the programmable logic (PL) side. Specifically, the 2048-point FFT module, the associated data-buffering FIFOs, and the pipelined digital notch filters all maintain relatively low consumption of lookup tables (LUTs), flip-flops (FFs), block RAMs (BRAMs), and DSP slices. Compared with the conventional 16,384-point large-size FFT architecture, the proposed design not only reduces BRAM storage consumption by nearly eight times, but also significantly decreases the power consumption of DSP multiply–accumulate units operating under high-frequency clock conditions. The detailed post-synthesis hardware resource utilization is summarized in Table 2.

The experimental results demonstrate that the proposed 2048-point optimized architecture achieves a substantial reduction in overall hardware resource utilization while maintaining the same optimal estimation accuracy. In particular, the utilization of block RAM (BRAM), which constitutes a critical bottleneck for on-chip memory resources, is reduced by up to 78.6%. This result not only verifies the low-complexity characteristic of the proposed algorithm, but also highlights its significant practical value for deployment in SWaP-constrained (size, weight, and power) tactical anti-jamming platforms.

End-to-end latency characterization. In real-time tracking systems under high-dynamic interference conditions, the physical processing latency is a critical factor determining whether stable closed-loop tracking can be maintained. By instrumenting FPGA hardware logic with test flags (Flags) and conducting oscilloscope-based measurements, the proposed system achieves an overall end-to-end response latency of approximately 140 μs under a 30.72 MHz RF sampling rate and a 100 MHz PL clock frequency.

The measured latency can be decomposed into four primary stages: RF front-end acquisition and transmission latency, PL-side computational latency, PS-side processing and communication latency, and digital notch filter steady-state establishment latency.

RF front-end acquisition and transmission latency. The dominant contribution in this stage arises from RF data acquisition. Given a sampling rate of 30.72 MHz, the time required to collect 2048 samples is approximately 66.67 μs. Considering subsequent data transfer and buffering overhead in the front-end pipeline, the total RF front-end latency is approximately 70 μs.

PL-side computational latency. This stage includes FFT processing and the hardware implementation of the three-spectral-line RIFE-based initial estimation. Operating at a 100 MHz clock frequency, the PL architecture enables rapid computation and allows the initial coarse alignment to update the notch filter in the first iteration, thereby achieving fast suppression of interference. According to ILA (Integrated Logic Analyzer) flag measurements, the latency from signal reception in the PL domain to data transmission to the PS domain is 23.87 μs.

PS-side processing and communication latency. This stage comprises processor interrupt handling, acquisition of coarse alignment results via the AXI bus, execution of two Newton-based refinement iterations, and writing the updated filter coefficients back to PL registers. Based on ILA timestamped flag analysis, the latency from PS invocation to return of updated parameters is 30.46 μs.

Finally, the steady-state establishment delay of the digital notch filter is considered. This delay is defined as the transient transition time required for its magnitude response to achieve at least 90% of the steady-state suppression depth. For a digital notch filter, the system’s transient response is determined by the poles of its denominator. The envelope of the transient response component, $y_{env}\left[n\right]$, strictly follows an exponential decay law:

(34) $$y_{env}\left[n\right]=\left|C\right|\cdot p^{n}$$

By taking the logarithm of both sides of Equation (34), the number of discrete sampling points required to reach this state can be determined. Given the pole radius p=0.995 and the requirement to achieve 90% of the steady-state magnitude response (which corresponds to a residual transient error of 0.1), the necessary number of sampling points, *n*, can be calculated as follows:

(35) $$n=\frac{\ln(0.1)}{\ln(0.995)}\approx \frac{-2.3025}{-0.00501}\approx 459.3$$

Considering the system’s data sampling rate of 30.72MHz, the resulting physical system delay is approximately 14.97 μs.

In summary, the complete slow-loop processing chain, including fine parameter calibration, exhibits an end-to-end latency of approximately 139 μs. The interaction between system latency and the dynamics of the interference signal directly determines the worst-case transient frequency tracking lag.

Taking a frequency sweep rate of 5 MHz/s as an example, the maximum tracking lag is strictly bounded to approximately 700 Hz. For an ultra-narrow notch filter with pole radius p=0.995, such a 700 Hz relative deviation only shifts the interference component away from the exact null, placing it within the transition (side-lobe) region of the filter’s stopband. Nevertheless, the residual attenuation provided in this region remains sufficient to suppress the interference power within the linear operating range of the receiver automatic gain control (AGC), thereby preventing receiver loss-of-lock and ensuring robust anti-jamming performance.

In contrast, if a conventional 16,384-point FFT-based scheme is adopted to achieve comparable accuracy, the RF data buffering stage alone introduces a latency of approximately 533 μs, resulting in a total system latency well exceeding 600 μs. Under the same dynamic condition of 5 MHz/s, the resulting tracking lag exceeds 3000 Hz, causing the interference signal to drift completely outside the effective stopband of the digital notch filter, ultimately leading to a collapse of the dynamic tracking process.

These results not only validate the necessity of the proposed architecture, but also highlight that reducing FFT size via Newton-based iterative refinement is a key engineering strategy for breaking the fundamental hardware latency bottleneck and significantly enhancing system robustness under high-dynamic interference conditions.

## 4. Performance Test of Anti-Jamming Platform

This section aims to comprehensively evaluate the overall performance of the adaptive anti-jamming platform proposed in this paper, which is built upon the Newton-iteration-enhanced three-spectral-line RIFE algorithm and a PS–PL heterogeneous architecture. In the complex electromagnetic environment encountered in modern satellite navigation systems, an effective anti-jamming solution must not only achieve high-precision frequency estimation of interference signals, but also ensure ultra-low response latency at the microsecond level. Accordingly, the experimental design is rigorously centered on two key performance metrics: frequency estimation accuracy and hardware real-time response capability.

To systematically assess both the theoretical advantages and engineering feasibility of the proposed platform, the experimental evaluation is organized into three progressively structured scenarios: a Fixed-Frequency Interference Test, a Sweep-Frequency Interference Test, and a GNSS Closed-Loop Positioning Performance Test.

The Fixed-Frequency Interference Test evaluates the system’s robustness and suppression capability against stable single-tone interference over a range of frequencies. By comparatively analyzing the notch depth and residual spectral energy, this test quantitatively characterizes the absolute accuracy of frequency estimation under static conditions.

The Sweep-Frequency Interference Test is designed to emulate highly dynamic and non-stationary jamming environments. By injecting rapidly varying swept-frequency interference signals, this experiment assesses the system’s frequency tracking capability, dynamic notch performance, and overall response latency of the underlying fast-loop processing chain during time-varying conditions.

To further validate the effectiveness of the proposed algorithm in practical deployment scenarios, a closed-loop experimental platform integrating real signal sources and a commercial STA8135 receiver (STMicroelectronics, Geneva, Switzerland) is established. From a system-level perspective, this test demonstrates the platform’s ability to maintain stable carrier-to-noise density ratios (C/N_0_) and continuous positioning solutions under severe jamming-to-signal ratio conditions.

### 4.1. Fixed-Frequency Interference Experiment

**Figure 7:**
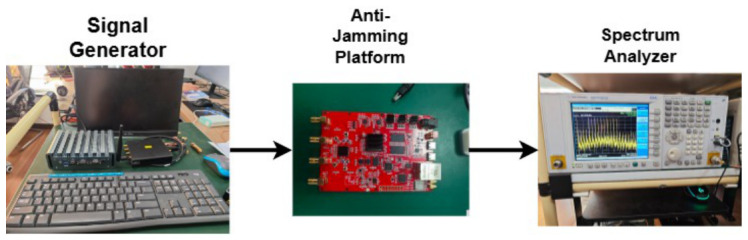
Hardware experimental platform.

The fixed-frequency jamming Experiment assesses the system’s robustness against stable single-tone interference across various frequencies, focusing primarily on frequency estimation accuracy. The experimental setup is illustrated in Figure 7. A USRP B210 platform (Ettus Research, Santa Clara, CA, USA) acts as the signal source, utilizing the GNU Radio software version 3.10-defined radio (SDR) framework to generate and transmit both single-tone and sweep signals. The generated signal is fed into the RX1 input of the anti-jamming platform, which subsequently feeds its processed output into a spectrum analyzer for residual signal energy measurement.

As detailed previously, the experimental setup utilizes an AD9361 RF front-end configured with a 30.72 MHz sampling rate and a 15 MHz reception bandwidth, enabling simultaneous coverage of the GNSS L1 and B1 bands. To emulate the varying frequency offsets encountered in practical applications, single-tone interference tests are conducted at 1 kHz intervals, originating from the L1 center frequency of 1575.42 MHz and comprising 20 distinct test iterations.

**Figure 8:**
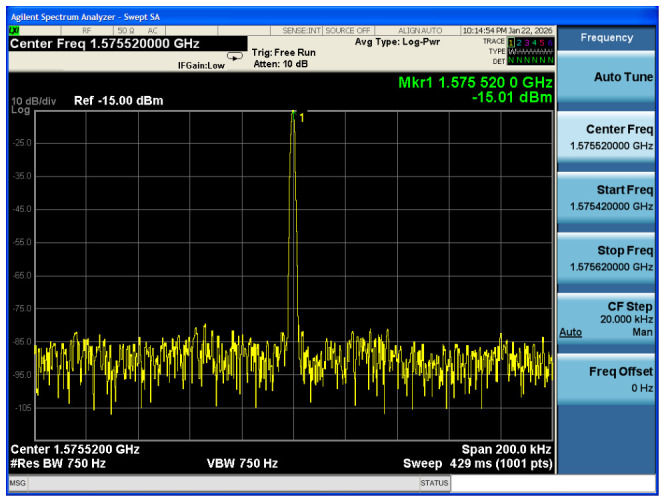
Raw single-tone interference.

**Figure 9:**
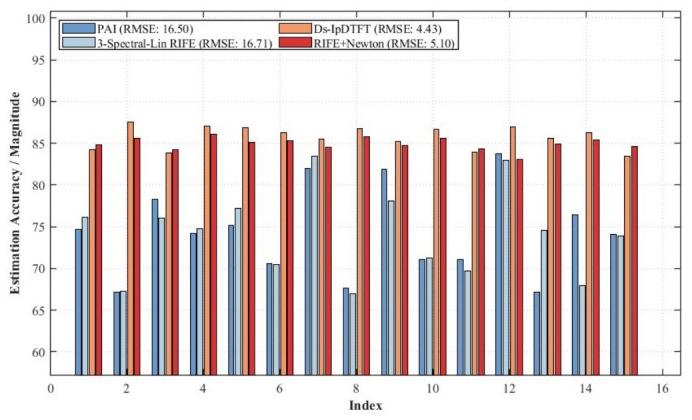
Results of single-tone interference experiment.

Figure 8 illustrates the spectrum of the original experimental jamming signal, acting as the system input, as displayed on the spectrum analyzer. As observed, the peak power of this original input signal is −15.01 dBm. In the subsequent experimental procedures, the center frequency of the original jamming signal is incrementally shifted to the right. The residual peak power, following interference suppression, is recorded for every 1 kHz shift, culminating in 15 sets of empirical data. This methodology is adopted because, under these conditions, the frequency offset induced by the FFT picket-fence effect during target signal identification is inherently random. Consequently, conducting these multiple trials enables the rigorous evaluation of the system’s authentic anti-jamming capability across diverse frequency offset scenarios. The experimental results are presented in Figure 9 and Table 3.

As illustrated in Figure 9 and Table 3, the experimental results clearly demonstrate the performance differences among four algorithms in a single-tone interference suppression scenario. The direct estimation methods, namely PAI and the three-spectral-line RIFE algorithm, exhibit evident limitations in suppression depth, achieving average interference attenuation levels of only 74.35 dB and 74.06 dB, respectively. In contrast, the proposed Newton-iteration-enhanced three-spectral-line RIFE significantly improves the suppression performance, reaching an average attenuation of 84.95 dB. This corresponds to an additional gain of approximately 10.9 dB over conventional methods, indicating a substantial enhancement in interference cancelation capability. It is worth noting that, although constrained by the requirement of ultra-low-complexity hardware implementation, the proposed method achieves a slightly lower average suppression level compared with the state-of-the-art Ds-IpDTFT algorithm (85.76 dB), with a marginal difference of only 0.81 dB. Such a negligible gap is generally considered insignificant in practical anti-jamming systems and does not lead to observable degradation in overall system performance.

Furthermore, conventional direct estimation methods exhibit significant performance fluctuations across repeated trials. The root mean square error (RMSE) of PAI and the three-spectral-line RIFE are as high as 16.50 and 16.71, respectively, indicating strong sensitivity to variations in initial frequency offset and resulting in highly unstable suppression performance. In contrast, the proposed cascaded Newton-refined algorithm substantially reduces performance variability, achieving an RMSE of 5.10, which is close to that of Ds-IpDTFT (4.43). This demonstrates excellent convergence behavior and robustness against perturbations. The results confirm that introducing Newton-based iterative refinement effectively mitigates the sensitivity to initial frequency offset in the coarse estimation stage, thereby providing consistently accurate frequency estimates across a wide range of initial conditions.

In summary, under single-tone interference conditions, the proposed algorithm achieves comprehensive improvements over conventional direct estimation methods in terms of both steady-state suppression depth and dynamic stability. Moreover, it attains near state-of-the-art performance comparable to Ds-IpDTFT, with only minimal sacrifice in extreme accuracy, thereby achieving a favorable trade-off among accuracy, robustness, and computational complexity.

### 4.2. Sweeping Frequency Interference Experiment

The chirp interference experiment is designed to evaluate the frequency tracking speed and dynamic notch performance of the proposed system under time-varying frequency conditions, with the objective of quantifying its response latency to interference signals.

The experimental configuration remains identical to that of the previous section, with all RF front-end parameters kept unchanged. Chirp signals with sweep rates of 2.5 MHz/s, 5 MHz/s, and 10 MHz/s are injected to emulate different levels of frequency dynamics.

A spectrum analyzer is employed to comprehensively evaluate the performance of four frequency estimation algorithms, namely the PAI-RIFE algorithm, the three-spectral-line RIFE algorithm, the proposed Newton-iteration-enhanced three-spectral-line RIFE, and the Ip-DFT algorithm. To enhance the dynamic performance of conventional algorithms, the FFT size of direct estimation methods was reduced in the experiments. After deployment on the hardware platform, a full performance assessment is conducted under identical experimental conditions.

**Figure 10:**
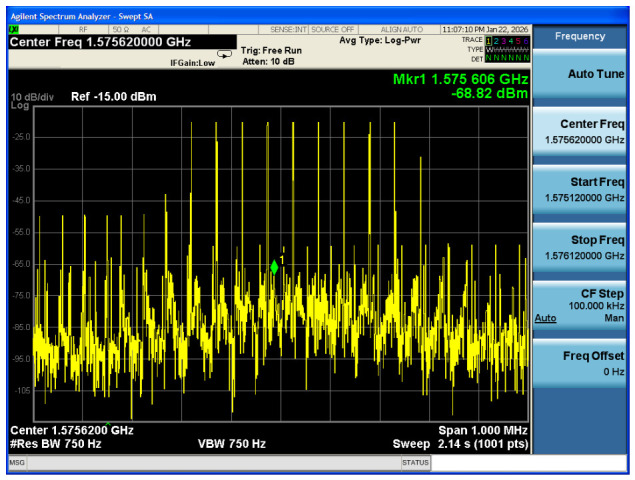
Raw sweep interference.

Figure 10 presents the spectrum of the original chirp signal. Due to the inherently high dynamic nature of chirp signals, the measured peak amplitude is highly sensitive to variations in the video bandwidth (VBW) setting. To ensure measurement consistency and accuracy, the VBW is strictly fixed at 750 Hz throughout the experiment. As illustrated in the figure, the peak spectral power of the original chirp signal is approximately −20 dBm.

**Figure 11:**
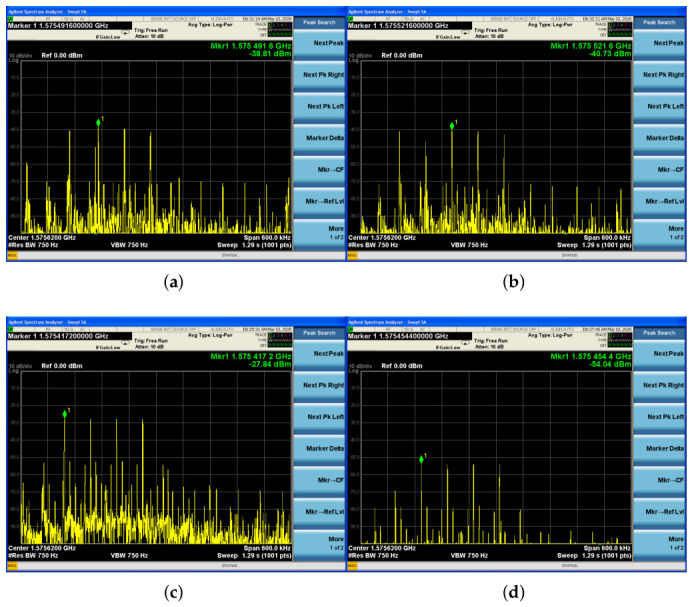
Experimental results under a 2.5 MHz/s chirp sweep. (**a**) Experimental results of the PAI-RIFE algorithm. (**b**) Experimental results of the three-spectral-line RIFE algorithm. (**c**) Experimental results of the Ds-IpDTFT algorithm. (**d**) Experimental results of the Newton-iteration-enhanced three-spectral-line RIFE algorithm.

Subsequently, the signal is processed using four different frequency estimation and interference suppression methods, namely the PAI-RIFE algorithm, the three-spectral-line RIFE algorithm, the IpDFT algorithm, and the proposed method. The corresponding interference suppression results under a 2.5 MHz/s sweep rate are shown in Figure 11.

Based on the experimental results in Figure 11, under a high-dynamic chirp interference scenario with a sweep rate of 2.5MHz/s, the differences in interference suppression performance among the evaluated algorithms clearly demonstrate the critical impact of processing latency on dynamic tracking capability. The experimental results indicate that the proposed Newton-iteration cascaded algorithm achieves the best performance, reaching an interference suppression level of approximately −35dB. This improvement is attributed to its extremely low computational latency enabled by a reduced FFT size and the quadratic convergence property of Newton iterations, requiring only two refinement steps. In contrast, the PAI-RIFE and conventional three-spectral-line RIFE algorithms exhibit lower processing latency; however, their performance is fundamentally limited by estimation accuracy constraints, resulting in suppression levels confined to the range of approximately −18dB to −20dB. Although the Ds-IpDTFT algorithm achieves high estimation accuracy under static conditions, its excessive computational burden due to multiple iterations introduces significant tracking latency. This leads to pronounced frequency tracking lag, causing the interference component to drift outside the effective stopband of the notch filter, and ultimately resulting in a degraded suppression performance of approximately −7 dB.

**Figure 12:**
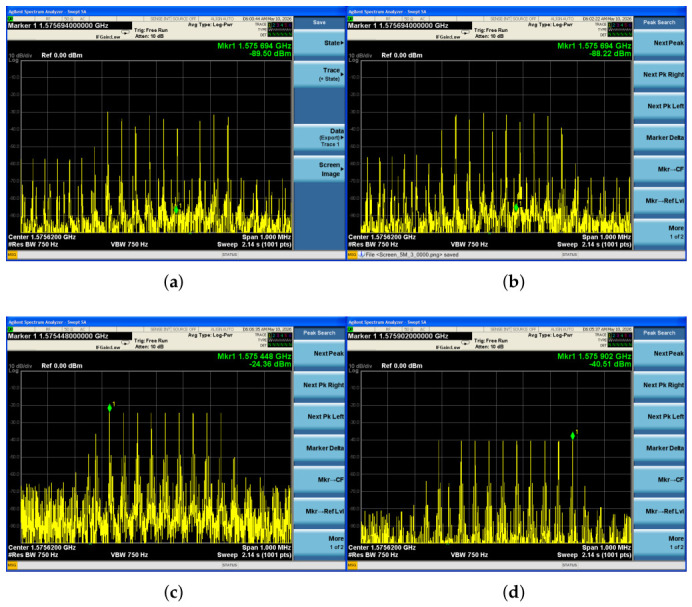
Experimental results under a 5 MHz/s chirp sweep. (**a**) Experimental results of the PAI-RIFE algorithm. (**b**) Experimental results of the three-spectral-line RIFE algorithm. (**c**) Experimental results of the Ds-IpDTFT algorithm. (**d**) Experimental results of the Newton-iteration-enhanced three-spectral-line RIFE algorithm.

As shown in Figure 12, under a higher chirp sweep rate of 5 MHz/s, the performance differences among the evaluated algorithms are further amplified, revealing the limitations of high-iteration methods in real-time systems. The experimental results indicate that the Ds-IpDTFT algorithm suffers from a severe real-time bottleneck due to its excessive computational complexity. As a result, its interference suppression capability degrades significantly to approximately −4dB, leading to an almost complete loss of tracking functionality under high-dynamic conditions. In contrast, the proposed Newton-iteration-enhanced algorithm benefits from its quadratic convergence property, which substantially reduces the closed-loop response time. Even under extreme chirp dynamics, it is still able to achieve an effective suppression level of approximately −21dB. Meanwhile, the PAI-RIFE and three-spectral-line RIFE algorithms exhibit comparable performance, both providing interference suppression levels of approximately −14 dB.

**Figure 13:**
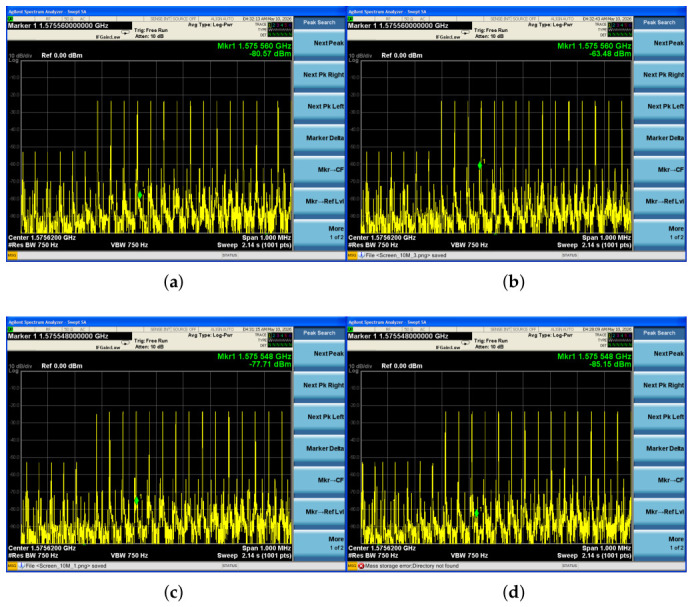
Experimental results under a 10 MHz/s chirp sweep. (**a**) Experimental results of the PAI-RIFE algorithm. (**b**) Experimental results of the three-spectral-line RIFE algorithm. (**c**) Experimental results of the Ds-IpDTFT algorithm. (**d**) Experimental results of the Newton-iteration-enhanced three-spectral-line RIFE algorithm.

As illustrated in Figure 13, when the chirp sweep rate is further increased to 10MHz/s, all evaluated algorithms exhibit severe degradation in interference suppression performance, indicating that the system has approached the dynamic tracking limit. Under such extreme frequency dynamics, the suppression capability of all four methods deteriorates substantially, and effective interference mitigation can no longer be maintained.

In this study, chirp sweep rates ranging from 2.5 MHz/s to 10 MHz/s are employed to comprehensively evaluate the interference suppression performance of different algorithms under high-dynamic conditions.

The experimental results demonstrate that the PAI-RIFE and conventional three-spectral-line RIFE algorithms are capable of maintaining basic real-time responsiveness. However, their sub-bin frequency estimation accuracy is fundamentally constrained, preventing precise alignment between the notch filter zeros and the interference center frequency. Consequently, the residual interference after suppression exhibits significant power fluctuations. Such unstable suppression behavior can induce severe fluctuations in the carrier-to-noise density ratio ($C/N_{0}$) within the receiver tracking loop, thereby degrading receiver lock stability.

Although the Ds-IpDTFT algorithm achieves excellent performance under static single-tone interference conditions, it almost completely loses its suppression capability in chirp interference scenarios. This degradation originates from its reliance on multiple iterative search procedures to achieve high estimation accuracy, which introduces substantial computational latency. The resulting tracking lag causes the generated notch suppression region to continuously fall behind the instantaneous interference frequency, allowing the interference signal to pass directly through the filter stopband. These results confirm the limitations of conventional high-precision iterative algorithms in real-time anti-jamming applications.

In contrast, the proposed Newton-iteration-enhanced cascaded algorithm successfully achieves an effective balance between estimation accuracy and system response speed by significantly reducing the FFT size to 2048 points and exploiting the quadratic convergence property of Newton iterations. Experimental results show that the proposed method is still capable of maintaining an effective suppression depth of approximately −21dB at a chirp sweep rate of 5MHz/s.

In summary, the proposed algorithm not only overcomes the accuracy limitations of direct estimation methods, but also breaks through the high-latency bottleneck associated with conventional iterative approaches. As a result, effective real-time suppression of chirp interference is achieved, demonstrating strong robustness for satellite navigation receivers operating in highly dynamic and complex electromagnetic environments.

### 4.3. Closed-Loop Test of GNSS Positioning Performance

**Figure 14:**
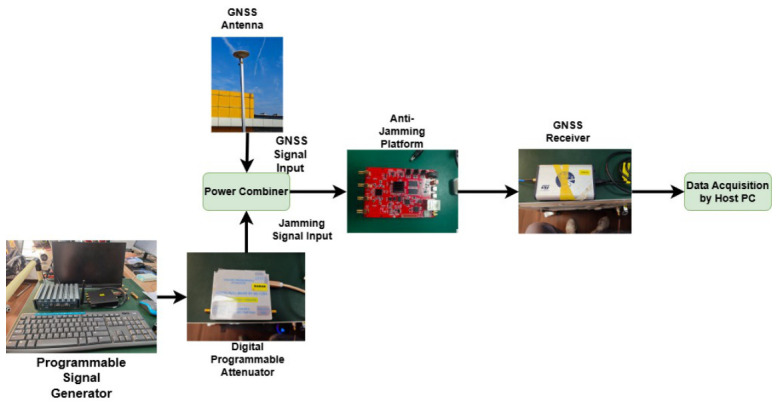
Closed-loop test hardware experimental platform.

To further validate the effectiveness of the proposed algorithm in practical engineering applications, a comprehensive test platform was established integrating the anti-jamming hardware with a GNSS receiver. The configuration of this experimental setup is illustrated in Figure 14.

The interference signal is generated using a software-defined radio (SDR) platform with an initial power level of −20dBm, consistent with the experimental configuration adopted in the previous tests. The chirp sweep range covers the entire L1 band. The signal is first passed through a digitally controlled attenuator governed by the host computer, and is then injected into an RF combiner, where it is superimposed onto the authentic satellite signal. The combined RF signal is subsequently fed into the front-end of the proposed anti-jamming platform, which performs interference mitigation before forwarding the processed signal to the GNSS receiver. Finally, the host computer records and visualizes the telemetry data output from the receiver.

To ensure experimental rigor and isolate the performance evaluation, single-tone interference is applied exclusively to the L1 band. Meanwhile, to eliminate the influence of multi-band-assisted positioning, the receiver is strictly configured to operate solely in the L1 band, with all other frequency bands disabled.

The data acquisition procedure begins with the collection of baseline reference data under interference-free conditions. Subsequently, the jamming power is increased incrementally at 30 s intervals. This gradual approach avoids abrupt tracking loop failures that may arise from sudden power surges, which could otherwise prevent the receiver loops from adapting in a timely manner. In addition, the controlled increment strategy mitigates the occurrence of “false lock” phenomena, where the receiver temporarily maintains positioning before ultimately losing lock, thereby ensuring that the tracking loops reach a steady state for reliable measurement.

During the measurement process, the host computer simultaneously records the positioning solutions and the signal-to-noise ratio of each tracking channel. To reduce data variability caused by transient satellite loss-of-lock and reacquisition events, the average of the four highest SNR values is computed and adopted as the representative SNR for the final results and visualization. In this experiment, three methods are selected for performance comparison, including the PAI-RIFE algorithm, the three-spectral-line RIFE algorithm, and the proposed three-spectral-line RIFE combined with Newton iterative refinement. The Ip-DFT algorithm is excluded from this evaluation due to its poor performance under chirp interference conditions observed in the previous experiment.

**Figure 15:**
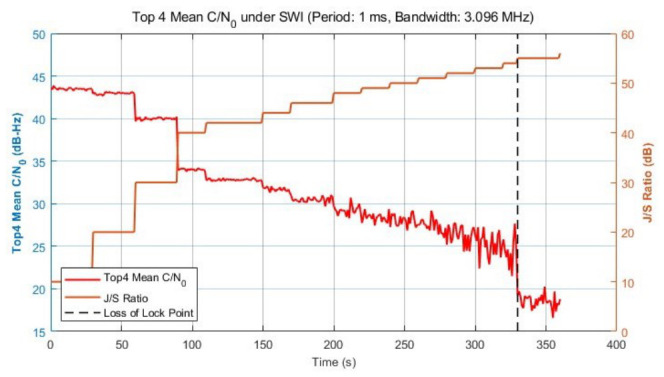
Dry interference test of receiver without interference suppression.

As illustrated in Figure 15, the positioning results remain unaffected during initial stages. The satellite signal-to-noise ratio (SNR) jitter progressively increases with rising interference intensity. While positioning remains achievable at a jamming-to-signal ratio of 53 dB, the system experiences lock loss with the Top4 satellites. Although the total satellite count stays above four to meet positioning requirements, the frequent switching of these top satellites causes significant jitter. When the jamming-to-signal ratio further increases to 54 dB, the SNR decreases sharply due to fewer than four satellites remaining, resulting in lock loss and failure to re-establish the connection.

**Figure 16:**
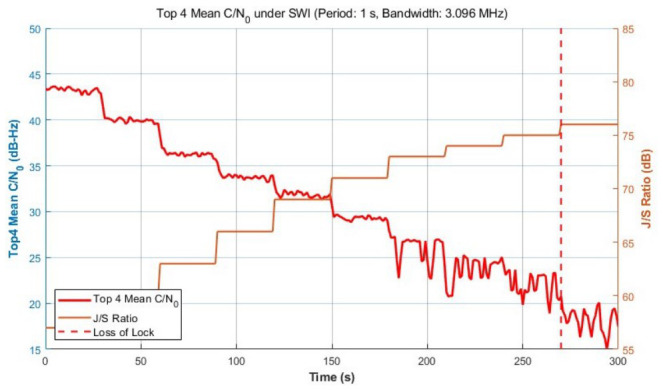
Anti-jamming test of receiver with PAI-RIFE algorithm.

The anti-jamming performance of the PAI-RIFE algorithm is illustrated in Figure 16. After applying the proposed suppression strategy, the carrier-to-noise density ratio ($C/N_{0}$) of the satellite signals exhibits an initial degradation and continues to decrease as the jammer-to-signal ratio (J/S) increases. When the J/S reaches approximately 72dB, noticeable fluctuations appear in the receiver tracking channels, indicating degraded tracking stability under strong interference conditions. Nevertheless, the receiver is still capable of maintaining marginal signal tracking to satisfy the minimum positioning requirement. However, when the J/S is further increased to 76dB, the receiver crosses the loss-of-lock threshold, causing the $C/N_{0}$ to rapidly drop below 20dB-Hz. As a consequence, the positioning solution completely fails.

**Figure 17:**
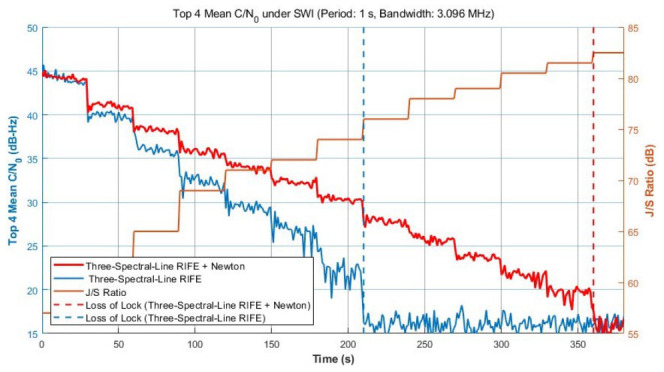
Anti-jamming test of receiver with Newton-iteration-enhanced three-spectral-line RIFE and three-spectral-line RIFE algorithm.

Figure 17 presents a comparative analysis of the anti-jamming performance between the conventional three-spectral-line RIFE algorithm and the proposed Newton-iteration-enhanced three-spectral-line RIFE algorithm. For the conventional three-spectral-line RIFE algorithm (blue curve), when the jammer-to-signal ratio (J/S) exceeds approximately 70 dB, the carrier-to-noise density ratio ($C/N_{0}$) begins to exhibit significant degradation accompanied by pronounced fluctuations. Under such conditions, the receiver tracking loop becomes highly unstable, and the algorithm eventually loses lock at a J/S level of 76 dB. In contrast, the proposed Newton-iteration-enhanced algorithm (red curve) demonstrates a substantially more stable performance profile. Although the $C/N_{0}$ still decreases gradually with increasing interference power, the corresponding trajectory remains significantly smoother, with reduced fluctuation amplitude. The proposed method is able to maintain stable signal tracking until a critical J/S level of 82 dB, at which point complete loss of lock occurs. This direct comparison clearly highlights the effectiveness of the proposed Newton-iteration refinement strategy. By refining the coarse initial frequency estimates, the proposed architecture effectively suppresses dynamic tracking jitter and significantly extends the overall anti-jamming margin of the receiver by approximately 6 dB under chirp interference conditions.

### 4.4. Experiment Summary

A comprehensive evaluation of the proposed Newton-iteration-enhanced three-spectral-line RIFE algorithm and the PS–PL heterogeneous anti-jamming platform is conducted through three progressive experiments, namely fixed-frequency interference tests, chirp interference tests, and GNSS closed-loop positioning experiments. The main conclusions are summarized as follows.

Under static narrowband interference conditions, the proposed method achieves an average residual interference power of −84.95dBm, outperforming conventional direct estimation methods (PAI-RIFE and three-spectral-line RIFE) by approximately 10.9 dB. It also approaches the performance of the state-of-the-art Ds-IpDTFT algorithm, with only a marginal difference of 0.81 dB, while significantly reducing performance variability (RMSE = 5.10 compared to values exceeding 16 for direct estimation methods). These results confirm that the Newton refinement stage effectively mitigates sensitivity to fractional frequency offsets and the fence effect, enabling consistent, deep, and stable notch filtering.

Under time-varying interference conditions, the proposed algorithm achieves a favorable trade-off between estimation accuracy and processing latency. By reducing the FFT size to 2048 points and exploiting the quadratic convergence property of Newton iterations (requiring only two refinement steps), the proposed method achieves approximately −35dB suppression at a 2.5 MHz/s sweep rate and −21dB at 5 MHz/s. In contrast, although the Ds-IpDTFT algorithm provides high accuracy under static conditions, its excessive computational latency leads to significant performance degradation under fast chirp dynamics. Direct estimation methods (PAI-RIFE and three-spectral-line RIFE) maintain low latency but are fundamentally limited by estimation accuracy, achieving suppression levels between −14dB and −20dB. When the sweep rate increases to 10 MHz/s, all algorithms approach the dynamic tracking limit, highlighting the severity of the test scenario.

On the practical receiver platform, the proposed method significantly extends the anti-jamming margin compared with direct estimation approaches. The receiver employing the proposed method maintains stable positioning and carrier-to-noise density ratio tracking up to a jammer-to-signal ratio (J/S) of 82 dB, whereas direct estimation methods lose lock at approximately 76 dB, corresponding to an improvement of about 6 dB. Moreover, the proposed method exhibits smoother trajectories and reduced fluctuations, further demonstrating its robustness under strong interference and dynamic operating conditions.

## 5. Conclusions

This paper presents a high-precision GNSS anti-jamming solution based on a Newton-iteration-enhanced three-spectral-line RIFE algorithm and a PS–PL heterogeneous implementation architecture. By combining a bias-reduced coarse estimator with an efficient iterative refinement scheme, the proposed method achieves high frequency estimation accuracy while significantly reducing the required FFT size.

Both simulation and experimental results demonstrate that the proposed algorithm outperforms conventional methods in terms of estimation accuracy, suppression depth, and robustness under dynamic interference conditions. In particular, under chirp interference conditions, the proposed method demonstrates significant advantages over both conventional direct estimation algorithms and iterative algorithms.

Furthermore, the hardware implementation validates the real-time capability of the proposed system, achieving microsecond-level processing latency on a Zynq-7000 platform. These results confirm that the proposed method is well-suited for practical deployment in next-generation GNSS anti-jamming systems.

Future work will focus on extending the proposed framework to multi-tone and wideband interference scenarios, as well as further optimizing the hardware architecture for lower power consumption and higher integration.

## Figures and Tables

**Table 1:** Comparison of different frequency estimation algorithms.

Algorithm	Estimation Accuracy	Computational Complexity	Response Speed
Direct Estimation Algorithms (e.g., Improved RIFE)	Relatively Low	Low	Relatively Fast
Traditional Iterative Algorithms	High	High	Slow
Three-Spectral-Line RIFE with Newton Iteration	High	Relatively High	Fast

**Table 2:** Comparison of hardware resource utilization.

Hardware Resource	Conventional 16,384-Point Module	Proposed 2048-Point Module	Saving Rate	Overall System Utilization
BRAM	44.5	9.5	78.6%	15.5
LUTs	5268	4061	22.9%	15,250
DSPs	21	17	19.0%	49
FFs	0	0	0%	25,225

**Table 3:** Statistical analysis of different frequency estimation algorithms.

Algorithm	Mean (dBm)	Standard Deviation (dBm)	95% Confidence Interval (dBm)
PAI-RIFE	73.97	5.54	[70.90, 77.04]
Conventional RIFE	74.24	5.22	[71.35, 77.12]
Ds-IpDTFT	85.83	1.16	[85.18, 86.47]
RIFE+Newton	84.95	0.82	[84.50, 85.40]

## Data Availability

The data presented in this study are available on request from the corresponding author.
